# 3D Alternating Direction TV-Based Cone-Beam CT Reconstruction with Efficient GPU Implementation

**DOI:** 10.1155/2014/982695

**Published:** 2014-06-19

**Authors:** Ailong Cai, Linyuan Wang, Hanming Zhang, Bin Yan, Lei Li, Xiaoqi Xi, Min Guan, Jianxin Li

**Affiliations:** ^1^National Digital Switching System Engineering & Technological R&D Centre, Zhengzhou, Henan 450002, China; ^2^Henan Province People's Hospital, Zhengzhou 450002, China

## Abstract

Iterative image reconstruction (IIR) with sparsity-exploiting methods, such as total variation (TV) minimization, claims potentially large reductions in sampling requirements. However, the computation complexity becomes a heavy burden, especially in 3D reconstruction situations. In order to improve the performance for iterative reconstruction, an efficient IIR algorithm for cone-beam computed tomography (CBCT) with GPU implementation has been proposed in this paper. In the first place, an algorithm based on alternating direction total variation using local linearization and proximity technique is proposed for CBCT reconstruction. The applied proximal technique avoids the horrible pseudoinverse computation of big matrix which makes the proposed algorithm applicable and efficient for CBCT imaging. The iteration for this algorithm is simple but convergent. The simulation and real CT data reconstruction results indicate that the proposed algorithm is both fast and accurate. The GPU implementation shows an excellent acceleration ratio of more than 100 compared with CPU computation without losing numerical accuracy. The runtime for the new 3D algorithm is about 6.8 seconds per loop with the image size of 256 × 256 × 256 and 36 projections of the size of 512 × 512.

## 1. Introduction 

Recently, iterative image reconstruction (IIR) algorithms [1–6], especially compressive sensing (CS) [7–10] based ones, have been developed for X-ray computed tomography (CT). As is widely known, CS based IIR algorithms can provide much higher image quality than the popular Feldkamp-Davis-Kress algorithm [11] (FDK) under sparse views. Constrained total variation (TV) based methods obtain impressive results for sparse view reconstruction in CT imaging [3, 12]. Although theoretical researches show that IIR possesses great advantages over analytical ones in image quality, it is still far from being put into practical use due to the expensive computation cost, especially for cone-beam computed tomography (CBCT). Fast image reconstruction is often required in clinical use to reduce the waiting time for the patient. Reconstruction speed is even more critical in real-time imaging applications, such as cardiac CBCT or online therapy.

Researchers in both optimization theory and hardware acceleration have made lots of progresses, aiming at developing more robust and efficient methods. The development of TV minimization indicates that the alternating direction method (ADM) [12, 13] can provide relatively better results. The representative algorithms using ADM are Lagrangian function based ones [14] and split Bregman method [15]. The two ADM based methods are equivalent under linear constraints. Both of the two kinds of optimization methods have been applied in CT reconstructions [12, 13, 16]. From another point of view, CBCT reconstruction can be regarded as an instance of high-performance computing [17]. Therefore, parallel processing can serve as an acceleration technique.

Originally designed for accelerating the computer graphics computation, the graphics processing unit (GPU) has emerged as a versatile platform for running massively parallel computation [18–21]. GPU provides clear advantages for CBCT image reconstruction: high memory bandwidth, high computation throughput, support for floating-point arithmetic, low price cost, and friendly programming interface. The acceleration of the filtered back-projection type algorithms using GPU represents a classic implementation of a nongraphics application on dedicated graphics hardware [22]. With the development of compute-specific APIs, CBCT reconstruction was accelerated using Brook [23] and CUDA [24–26]. In CUDA, FDK acceleration mainly focuses on back-projection making use of techniques of thread assigning, memory optimization, in-built arithmetic instructions, and so on [25, 26]. However, parallel processing for IIR algorithms meets new issues because these algorithms are fundamentally sequential. GPU acceleration needs sufficiently parallel workload [27]. Therefore, algorithms with minimal computation within each loop are not proper for GPU implementation. For instance, the algebraic reconstruction technique (ART) is not suitable for the GPU because each loop only processes a single beam. A more suitable algorithm is simultaneous ART (SART), which updates the image after the back-projection of an entire projection view. Several other iterative algorithms were also adapted to the GPU [28–30], including total variation reconstruction [31].

This paper proposes an efficient 3D IIR algorithm based on alternating TV minimization method for CBCT reconstruction based on GPU acceleration. An inexact ADM iteration using local linearization and proximity technique is adopted to avoid the pseudoinverse calculation. The experiments using both simulation and real CT data prove that the proposed algorithm for CBCT is both fast and accurate. The paper is outlined as follows.  Section 1 briefly discusses the incomplete data CBCT reconstruction problems and related works.  Section 2 shows the new method in detail and its parallelization analysis. The CUDA implementation and experiments on both simulation and real data results are introduced and shown in Section 3. Finally, Section 4 brings a brief discussion and conclusion.

## 2. Methods

A CBCT scanning system mainly consists of an X-ray source, interested object, and a flat panel detector. From a discrete to discrete point of view, the image system can be modeled as the following linear system:

(1) $$\begin{array}{c} p=Wf, \end{array}$$

where vector *p* ∈ *R* ^*N*_rays_^ has a length of *N*_rays_ which is the vectorization of the projection data; the vector *f* ∈ *R* ^*N*_voxels_^ has a length of *N*_voxels_ which stands for the discrete vectorized form of the object function. Matrix *W* ∈ *R* ^*N*_rays_×*N*_voxels_^ models the imaging system which has *N*_rays_ rows and *N*_voxels_ columns. In this work, the value in the system matrix is modeled using the ray intersection length with the cubic voxel. For incomplete angle problem, (1) is always undersampled and ill-conditioned. The CS theory indicates that the linear system can achieve exact solution under certain sparse representation by the following L1-norm minimization:

(2) $$\begin{array}{c} f^{*}=\arg\;\min{||\Psi \left(f\right)||}_{1},\\ \text{s}.\text{t}.\;p=Wf. \end{array}$$

For CT images, it is always the case that most of the images have very sparse gradient-magnitude images (GMI) [3]. It is a good tool to use GMI for CS based image reconstruction which is the origination of the famous TV-based algorithms.

### 2.1. Review of Alternating Direction TV Minimization Reconstruction

First of all, a brief review of alternating direction TV minimization reconstruction (ADVTM) [12] algorithm is carried out for the completeness of this paper. Apply the TV regularization to (1); then we will get the constrained TV minimization reconstruction model. Here, we use the anisotropic TV for CBCT reconstruction; that is, ||Ψ(*f*)||_1_ = ||*f*||_*TV*_≜∑_*j*_||*D*_*j*_ *f*||_1_, *j* = 1,2, 3. Here, *D*_1_, *D*_2_, and *D*_3_ stand for the differential operator in *X*, *Y*, and *Z* directions. The unconstrained form of (2) can be written as

(3) $$\begin{array}{c} \min\frac{1}{2}{||p-Wf||}^{2}+\rho \underset{j}{\sum }{||D_{j}f||}_{1}, \end{array}$$

where *ρ* stands for the penalty factor. Let *D*_*j*_ *f* = *z*_*j*_; equation (3) can also be transformed as

(4) $$\begin{array}{c} \min\frac{1}{2}{||p-Wf||}^{2}+\rho \underset{j}{\sum }{||z_{j}||}_{1}. \end{array}$$

The corresponding Lagrangian function of the above problem is

(5) min⁡LA(f,z,u)=min⁡12||p−Wf||2+∑j(ρ||zj||1+λ2||Djf−zj+ujλ||2),

where *u*_*j*_ ∈ *R* ^*N*_voxels_^ is multiplier, and *λ* ∈ *R* is the factor for square formation. Under the ADM framework, splitting the variables *f* and *z*, we get the following iteration form:

(6) $$\begin{array}{c} f^{\left(k+1\right)}=\arg\underset{f}{\min}\left({||p-Wf||}^{2}+\lambda \underset{j}{\sum }{||D_{j}f-z_{j}^{\left(k\right)}+u_{j}^{\left(k\right)}/\lambda ||}^{2}\right),\\ z_{j}^{\left(k+1\right)}=\arg\underset{z_{j}}{\min}\left(2\rho {||z_{j}||}_{1}+\lambda {||D_{j}f^{\left(k+1\right)}-z_{j}^{}+u_{j}^{\left(k\right)}/\lambda ||}^{2}\right),\\ u_{j}^{\left(k+1\right)}=u_{j}^{\left(k\right)}+\lambda \left(D_{j}f^{\left(k+1\right)}-z_{j}^{\left(k+1\right)}\right). \end{array}$$

The minimization with respect to *z*_*j*_ has the following closed form solution:

(7) zj(k+1)=max⁡{|Djf(k)+uj(k)λ|−ρλ,0}sgn⁡(Djf(k)+uj(k)λ).

For the minimization with respect to *f*, the optimization is a quadratic function and set its derivative to 0:

(8) (λ∑jDjTDj+WTW)f =(WTp+λ∑jDjT(zj(k)−uj(k)λ)).

The basic idea to find the solution to the above equation is to calculate the pseudoinverse of *λ*∑_*j*_ *D*_*j*_^*T*^ *D*_*j*_ + *W* ^*T*^ *W*. Therefore, the exact solution for the (*k* + 1)th iteration of the above *f* subproblem is as in the following expression:

(9) f(k+1)=(λ∑jDjTDj+WTW)+ ×(WTp+λ∑jDjT(zj(k)−uj(k)λ)),

where *X* ^+^ stands for the Moore-Penrose pseudoinverse of matrix *X*. The update form of multipliers is

(10) $$\begin{array}{c} u_{j}^{\left(k+1\right)}=u_{j}^{k}+\lambda \left(D_{j}f^{\left(k+1\right)}-z_{j}^{\left(k+1\right)}\right). \end{array}$$

Therefore, the ADTVM algorithm has the following iteration form.

**Algorithm 1 .**

**While** “not converged,” *k* ← 0** Do**

1. Update *f* using *f* ^(*k*+1)^ = (*λ*∑_*j*_ *D*_*j*_^*T*^ *D*_*j*_+*W* ^*T*^ *W*)^+^(*W* ^*T*^ *p* + *λ*∑_*j*_ *D*_*j*_^*T*^(*z*_*j*_^(*k*)^ − *u*_*j*_^(*k*)^/*λ*));
2. Update *z* using *z*_*j*_^(*k*+1)^ = max⁡{|*D*_*j*_ *f* ^(*k*)^ + (*u*_*j*_^(*k*)^/*λ*)| − (*ρ*/*λ*), 0}sgn⁡(*D*_*j*_ *f* ^(*k*)^ + (*u*_*j*_^(*k*)^/*λ*));
3. Update *u* using *u*_*j*_^(*k*+1)^ = *u*_*j*_^*k*^ + *λ*(*D*_*j*_ *f* ^(*k*+1)^ − *z*_*j*_^(*k*+1)^);
4. *k* ← *k* + 1

**End Do**

The ADTVM algorithm use exact solutions for each subproblem at each iterative loop and it has the assurance of the convergence. The application of ADTVM algorithm for 2D reconstruction has already shown some impressive results [12].

### 2.2. The 3D Inexact Alternating Direction Reconstruction

It can easily be seen that the ADTVM reconstruction has a very simple iteration form, and its convergence property makes it a robust algorithm. However, let us take a more careful analysis of the above algorithm. In fact, it should be pointed out that the ADTVM iteration involves a very expensive calculation of the pseudoinverse for a huge matrix *λ*∑_*j*_ *D*_*j*_^*T*^ *D*_*j*_ + *W* ^*T*^ *W*. More seriously, the ADTVM may fail in cone-beam reconstruction for even a small scale of 3D data set, saying a cube having size of 256 × 256 × 256. Actually, it is impossible to have such huge memory to store the cone-beam system matrix for a personal computer. Consequently, for a cone-beam reconstruction problem, the ADTVM is actually not applicable for it cannot be implemented. In fact, methods that only use *W* and its transpose make sense in finding the solution to cone-beam reconstruction problems. Therefore, it is essential to develop a more practical and efficient algorithm for 3D reconstruction based on alternating direction method.

In this subsection, a practical alternating direction reconstruction using local linearization and proximity technique is proposed with GPU aided computation. In matrix computation theory [31], matrix with some special structures, such as diagonal matrixes or those which can be diagonalized by FFTs, can help in improving the calculation performance greatly. However, for the general matrix *W* in CBCT, *W* ^*T*^ *W* is neither diagonal nor FFT diagonalizable. We adopt an inexact strategy to tackle this subproblem of minimization for *f*. For minimization with respect to *f*, the fidelity term of ||*p*−*Wf*||^2^ in (6), that is, the term containing *W*, is linearized at the current point *f* ^(*k*)^ and its proximal form is

(11) $$\begin{array}{c} {||p-Wf||}^{2}\approx {||p-Wf^{\left(k\right)}||}^{2}+2g_{k}^{T}\left(f-f^{\left(k\right)}\right)+\frac{1}{\tau }{||f-f^{\left(k\right)}||}^{2}, \end{array}$$

where *g*_*k*_ = *W* ^*T*^(*Wf* ^(*k*)^ − *p*) is the gradient of ||*p*−*Wf*||^2^ at the current point of *f* ^(*k*)^, and *τ* > 0. Consequently, the subproblem of *f* can be converted into the following form:

(12) min⁡f||p−Wf(k)||2+2gkT(f−f(k)) +1τ||f−f(k)||2+λ∑j||Djf−zj(k)+uj(k)λ||2.

Set the derivative of the above quadratic function to 0, we get

(13) $$\begin{array}{c} \left(\frac{1}{\tau }I+\lambda \underset{j}{\sum }D_{j}^{T}D_{j}\right)f=c_{k}, \end{array}$$

where *c*_*k*_ = (1/*τ*)*f* ^(*k*)^ − *W* ^*T*^(*Wf* ^(*k*)^ − *p*) + *λ*∑_*j*_ *D*_*j*_^*T*^(*z*_*j*_^(*k*)^ − *u*_*j*_^(*k*)^/*λ*). Under the periodic boundary condition, ∑_*j*_ *D*_*j*_^*T*^ *D*_*j*_ is a block circulant matrix. Therefore, the coefficient matrix on the left hand side of (13) can be diagonalized by three-dimensional fast Fourier transform *F*_3_ via *F*_3_((1/*τ*)*I* + *λ*∑_*j*_ *D*_*j*_^*T*^ *D*_*j*_)*F*_3_^−1^ = *M*. Let Λ(*M*) = *J* ∈ *R* ^*N*_voxels_^, where Λ(*M*) = *J* means that *J* is composed by the elements on the diagonal of *M*. Apply 3D Fourier; transform both sides of (13); the solution of (13) can be computed efficiently by

(14) f(k+1)=F3−1 ×(F3(1τf(k)−WT(Wf(k)−p)     +λ∑jDjT(zj(k)−uj(k)λ))×J−1),

where the division of *A*/*B* is a component-wise operation. The new algorithm is implemented as the following list.

**Algorithm 2 .**

**While** “not converged,” *k* ← 0** Do**

1. Update *f* using *f* ^(*k*+1)^ = *F*_3_^−1^(*F*_3_((1/*τ*)*f* ^(*k*)^ − W^*T*^(*Wf* ^(*k*)^ − *p*) + *λ*∑_*j*_ *D*_*j*_^*T*^(*z*_*j*_^(*k*)^ −*u*_*j*_^(*k*)^/*λ*))/*J*),
2. Update *z* using *z*_*j*_^(*k*+1)^ = max⁡{|*D*_*j*_ *f* ^(*k*)^ + (*u*_*j*_^(*k*)^/*λ*)| − (*ρ*/*λ*), 0}  sgn⁡(Djf(k)+(uj(k)/λ));
3. Update *u* using *u*_*j*_^(*k*+1)^ = *u*_*j*_^*k*^ + *λ*(*D*_*j*_ *f* ^(*k*+1)^ − *z*_*j*_^(*k*+1)^);
4. *k* ← *k* + 1

**End Do**

It can easily be seen that the calculation of *f* ^(*k*+1)^ is closely related to *f* ^(*k*)^ which is different from that in Algorithm 1. Notably, the ADTVM involves the calculation of *W* ^*T*^ *W* and (*λ*∑_*j*_ *D*_*j*_^*T*^ *D*_*j*_+*W* ^*T*^ *W*)^+^ which can only be implemented based on storing the system matrix *W* beforehand. However, even for the occasion of 2D reconstruction, the system matrix is actually so tremendous that its pseudoinverse calculation is very time consuming. Furthermore, for 3D situation for ADTVM, there is no such a huge storage device which can accommodate such a big system matrix. Consequently, the pseudoinverse computation in ADTVM is very impractical or even impossible to be implemented for 3D reconstruction because of time and memory consumption. The new algorithm utilizes the linearization technique which ably avoids the bother of storing the system matrix. Moreover, the new method also averts the horrible computation of *W* ^*T*^ *W* and (*λ*∑_*j*_ *D*_*j*_^*T*^ *D*_*j*_+*W* ^*T*^ *W*)^+^. In addition, the involved FFT techniques can further improve the computation efficiency. These characteristics make the new algorithm an indispensable method for cone-beam image reconstruction based on the alternating direction method. The convergence property is guaranteed and discussed in detail in [32].

### 2.3. GPU Implementation

The related forward- and backward-projection operations in *W* ^*T*^(*Wf* ^(*k*)^ − *p*) has very high complexity for CPU computation. Generally, the forward-projection in Algorithm 2 can be defined as

(15) $$\begin{array}{c} p_{i}=\underset{j\in Q_{i}}{\sum }w_{i,j}f_{j}, \end{array}$$

where *f* is the attenuation coefficient, *w*_*i*,*j*_ is the value in the system matrix *W* at position of (*i*, *j*), and *Q*_*i*_ is the set containing all the indices of voxels that have nontrivial intersections with the beam *i*. Analogously, the backward-projection can be defined as

(16) $$\begin{array}{c} f_{j}=\underset{i\in Q_{j}}{\sum }w_{i,j}p_{i}, \end{array}$$

where *Q*_*j*_ is the set containing all the indices of beam that have nontrivial intersections with the voxel *j*. The iteration of Algorithm 2 is simple but convergent. Although there are only one forward- and one backward-projection operation in *W* ^*T*^(*Wf* ^(*k*)^ − *p*) at each iterative loop, these two operations can occupy most of the computation time. For more efficient implementation, more advanced hardware optimization besides local linearization and proximity technique in algorithm design should be taken into consideration. Traditional method for calculating the forward-projection is the ray tracing method proposed by Siddon. Siddon's algorithm uses a parametric line representation of the beam which makes the complexity of computing the intersection lengths of each beam with 3D domain still with respect to 1D line. For CBCT reconstruction, the system matrix is so tremendous that Siddon's algorithm is not suitable for computing both forward and backward projections simultaneously.

For efficient computation, a fast and parallel algorithm [33] for forward and backward projections is utilized in this paper. A brief review of this algorithm is given here and the detailed interpretation can be found in [33]. When computing the forward-projection, the 3D region of the object is divided into a group of planes in one direction according to the slope of the beam. This limits the number of the voxel intersected with the beam within quite few situations. Computing the length can be executed in parallel by each plane, which makes the calculation pretty efficient. When dealing with the backward-projection, the parallelization can be realized in parallel for each voxel. In finding the corresponding beams, a shadow region method is utilized [33].

**Figure 1:**
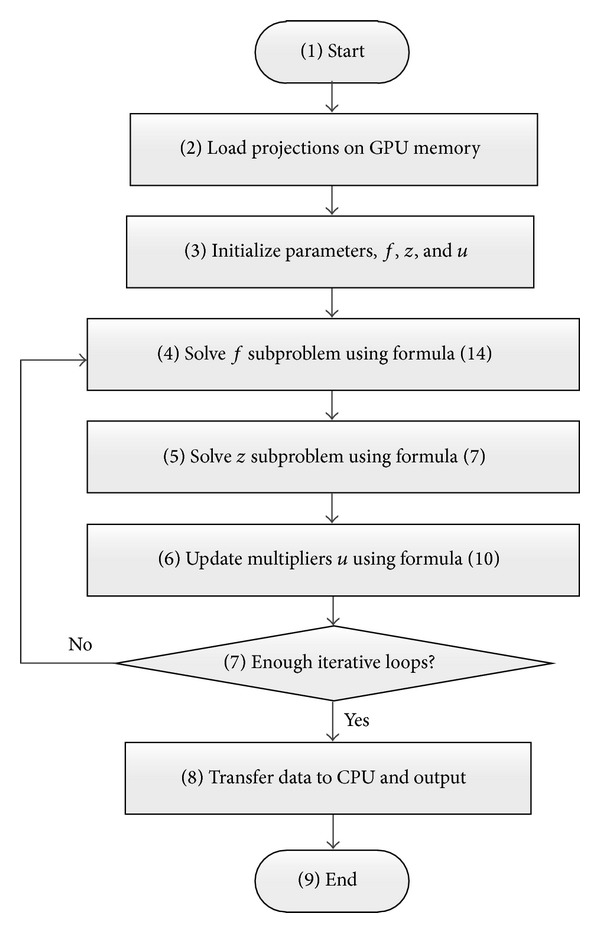
Flow chart of the proposed inexact alternating direction CBCT reconstruction algorithm. Blocks 4–6 correspond to 1–3 of Algorithm 2.

Although iterative algorithm is fundamentally sequential, the reconstruction algorithm we designed here for CBCT can be implemented efficiently with the aid of GPU considerably. The three update formulas can all be computed on GPU for speedup. The operations involved in the proposed method mainly include matrix-vector multiplications and vector additions. These operations include *D*_*j*_ *f*, *D*_*j*_^*T*^ *f*, *Wf*, and *W* ^*T*^ *f*. The operation of *D*_*j*_ *f* and *D*_*j*_^*T*^ *f* can be straightforwardly put into GPU calculation, with each thread computing the difference of a voxel. The most expensive calculation parts are *Wf* and *W* ^*T*^ *f* which stand for forward- and backward-projections. With the aid of the fast and parallel algorithm, the forward- and backward-projections can potentially be accelerated significantly. With the GPU aided computation, the flow chart of the proposed algorithm is shown in Figure 1.

## 3. Experiments

### 3.1. Computation Efficiency

To evaluate the performance of the CUDA aided implementation, we implement and test the operation of *D*_*j*_ *f*, *D*_*j*_^*T*^ *f*, *Wf*, and *W* ^*T*^ *f* both on CPU and on GPU. In addition, there are two data sets running on NVIDIA Tesla K20c. This GPU device has 2496 CUDA cores and 5120MB global memory. In the performance test, a 3D digital Moby mouse phantom in which the attenuation coefficient is in 0.0~1.0 is utilized. A single circle trajectory is utilized for cone-beam scanning. The source to axis distance is 30 cm, and the source to the center of the flat panel distance is 60 cm. The detector panel has a size of 12.8 cm × 12.8 cm. The phantom has a size of 6.4 cm × 6.4 cm × 6.4 cm. The projection data are collected by 36 equally angular views in 360 degrees.

In order to test the four operations under different data sets, two kinds of discretization are applied which is listed in Table 1. All the experiments are carried out on the workstation configured with dual cores of Intel Xeon CPU of E5-2620 @ 2.10 GHz (only one core was used) equipped with Tesla K20c. The time consumption for both CPU and different GPU is listed in Table 2 together with its speedup. All the time consumption is calculated by the statistical average of fifty times. The computation between CPU and GPU is expressed in root mean square error (RMSE) by $\text{RMSE}=\sqrt{\sum \left({\left(x_{\text{CPU}}-x_{\text{GPU}}\right)}^{2}/N\right)}$, where *N* stands for the total number of values; *x*_CPU_ and *x*_GPU_ stand for CPU results and GPU results, respectively. The RMSEs are listed in Table 3. The speedup in Table 2 shows that the acceleration strategy applied here can improve the performance greatly with GPU while Table 3 indicates that the numerical differences can be ignored.

### 3.2. Reconstruction Verifications

The reconstruction algorithm proposed in this paper is composed of *D*_*j*_ *f*, *D*_*j*_^*T*^ *f*, *Wf*, *W* ^*T*^ *f*, and a few vector additions and comparisons. In this subsection, reconstruction using both simulation data and real CT projections is carried out. The goal is to test the performance of the entire routine of the new algorithm and the image reconstruction quality. For the reconstruction of simulation data, the above data set of situation 2 in Section 3.1 is utilized. Its scanning configuration is the same as that in Section 3.1. For the real data reconstruction, projections of a medical head phantom are acquired with the cone-beam CT system which mainly consists of a flat panel detector (Varian4030E, USA) and an X-ray source (Hawkeye 130, Thales, France). The distance between source and the rotation axis of scanner is 678 mm and the distance between source and the detector is 1610 mm. The detector bin has a size of 0.508 mm × 0.508 mm. The projection size is 768 pixels × 432 pixels × 72 views. The size of reconstruction image is 384 voxels × 384 voxels × 216 voxels with 0.214 mm × 0.214 mm × 0.214 mm per voxel.

**Figure 2:**
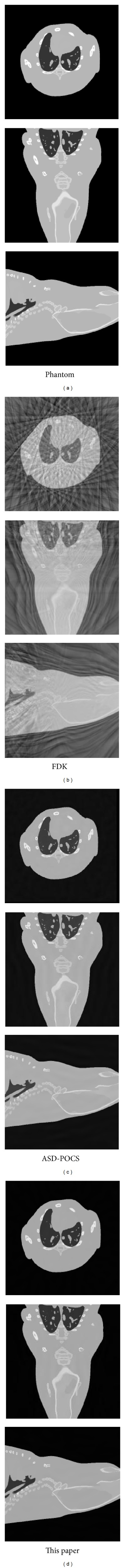
Digital phantom and the reconstructions for simulation data. The first column in the left is the phantom of 3D Moby mouse and the second, third, and fourth columns are the reconstructions of FDK, ASD-POCS, and the GPU accelerated new method. From the top row to the bottom row, there are slices of *z* = 31, *y* = 128, and *x* = 128 in phantom and the reconstructions.

**Figure 3:**
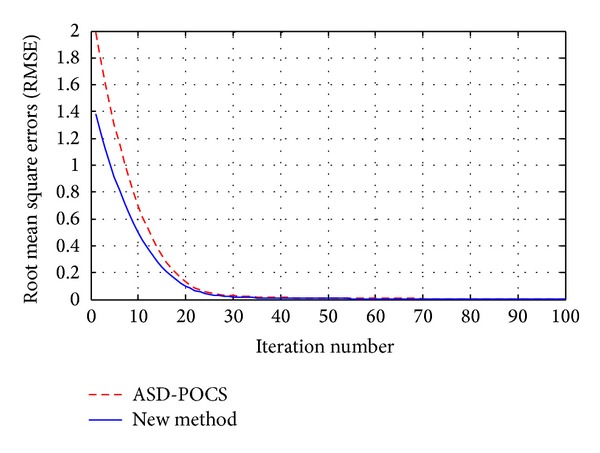
RMSEs versus iteration number for two algorithms.

**Figure 4:**
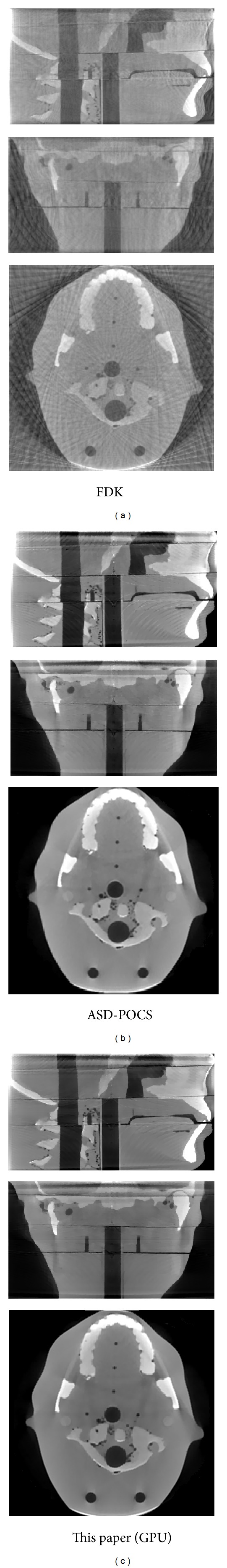
The reconstructions of real CT data experiments. The first, second, and third columns from the left to the right are results of FDK, ASD-POCS, and the GPU accelerated new method. From the top to the bottom row, there are results of slices of median sagittal section, central coronal section, and central transverse section.

In the reconstructions, the proposed algorithm is compared with FDK algorithm and the adaptive-steepest-descent-POCS (ASD-POCS) [3] algorithm. The parameters of the new method are empirically chosen as *τ* = 1, *ρ* = 1, and *λ* = 1. The parameters of ASD-POCS are the same as those in [3]. The iteration number of both simulation and real data reconstruction is 100. The simulation reconstruction results are shown in Figure 2, where a 3D slice of *z* = 31, *y* = 128, and *x* = 128 is presented. The RMSEs for ASD-POCS and the proposed method for simulation reconstruction are listed in Table 4. The convergence behavior of the new method for simulation is drawn in Figure 3. The real CT data experiments use 72 equally angular views. Reconstructions of the FDK, ASD-POCS, and the new method are shown in Figure 4.

The reconstruction results of FDK algorithm in Figures 2 and 4 suffer from streak artifacts so severely that the useful and detail structures are degraded or even lost. Therefore, the FDK reconstructions from 36 or 72 views can hardly be put into practical use. The ASD-POCS and the proposed algorithms provide satisfying image quality. The reconstruction results of these two methods do not show visible differences. Meanwhile, the RMSEs behavior of the new method in Figure 3 shows a robust convergence. The time consumptions for each reconstruction are listed in Table 5. From this table, it can be seen that the GPU device plays the key role for improving the reconstruction performance, and the acceleration ratio of the new method for GPU compared with CPU is about 106 for simulation and 120 for real data reconstruction, respectively. The reconstruction qualities of the proposed algorithm for simulation data and real data are both satisfying and are potential to be put into practical use.

## 4. Discussion and Conclusion

Reconstruction performance is an important issue and its acceleration is of crucial significance for iterative algorithms and this paper try to do some related work. This paper has proposed a GPU based alternating direction reconstruction method for cone-beam CT imaging. The new method utilizes a local point linearization and proximity strategy which avoids the calculation of pseudoinverse of matrix. The proximal process applied in the new algorithm makes it efficient and applicable for CBCT reconstruction using the ADM routine. Although the new method utilizes an approximate or inexact strategy to tackle the *f* subproblem, the reconstructions in both simulation and real data experiments show a robust convergence property. In fact, the augmented Lagrangian function (5) is expected to be minimized by solving *f* subproblem and *z* subproblem alternately. Therefore, solving these two subproblems accurately at each sweep may be unnecessary.

Furthermore, the advantages for the inexact strategy are not only avoiding the pseudoinverse computation, but also making the reconstructions able to efficiently be launched on GPU cards which is a key to improve the overall performance. Each calculation of the subproblems has some computation parts that can be executed in parallel on GPU cards, and the acceleration ratio for these parts can be rather high. The most important matter focuses on accelerating the most time consumption parts which will make an outstanding improvement. For the entire algorithm, the acceleration ratio is a little lower than that of each part which is mainly due to the serial computation parts running on CPU. The results in the reconstruction experiments show a considerable acceleration for the new algorithm while the reconstruction qualities are well kept.

The new algorithm applies a highly efficient technique to settle the difficulties faced by ADTVM in cone-beam imaging. Actually, the technique utilized in this paper is ingenious but necessary. The proximal method has no influence on the convergence of the algorithm. It is robust and its 3D reconstructions are both accurate and fast. Although the application presented here is circular cone-beam CT, it is clear that this algorithm and its GPU acceleration can be applied to other tomographic imaging modalities with linear system models. Future work will focus on further improving and optimizing the acceleration efficiency, so that the algorithm can be more practical for actual scanning systems.

## Figures and Tables

**Table 1:** Dataset for situations 1 and 2.

	Volume data	Projection data	Voxel size	Detector bin size
Situation 1	128 × 128 × 128	256 × 256 × 36	0.50 mm	0.50 mm
Situation 2	256 × 256 × 256	512 × 512 × 36	0.25 mm	0.25 mm

**Table 2:** Running time for related operation in the reconstruction (unit for time: seconds).

	Situation 1			Situation 2		
	CPU	GPU	Speedup	CPU	GPU	Speedup
*D*_*j*_ *f*	0.030000	0.0002312	129.76	0.398274	0.0026197	152.03
*D*_*j*_^T^ *f*	0.042977	0.0003432	125.22	0.529065	0.0032604	162.27
*Wf*	10.248665	0.058801	174.29	83.390976	0.452213	184.41
*W* ^T^ *f*	73.074226	0.399989	182.69	636.070923	3.151384	201.83

**Table 3:** RMSE of GPU computation for related operation.

	*D*_*j*_ *f*	*D*_*j*_^T^ *f*	*Wf*	*W* ^T^ *f*
Situation 1	0.5*E* − 6	0.4*E* − 6	2.3*E* − 6	1.7*E* − 6
Situation 2	0.2*E* − 6	0.1*E* − 6	1.5*E* − 6	0.9*E* − 6

**Table 4:** RMSEs for two reconstruction algorithms.

	20	40	60	80	100
ASD-POCS	0.1301	0.0150	0.0082	0.0058	0.0037
New method	0.1000	0.0102	0.0055	0.0045	0.0028

**Table 5:** Running time for simulation and real data experiments of the new algorithm.

	New method on CPU	New method on GPU	Acceleration Ratio
Simulation data	72114 seconds	681 seconds	105.89
Real data	3.733*E* + 5 seconds	3114 seconds	119.88
